# Exploring self-report and proxy-report quality-of-life measures for people living with dementia in care homes

**DOI:** 10.1007/s11136-019-02333-3

**Published:** 2019-10-23

**Authors:** Alys W. Griffiths, Sarah J. Smith, Adam Martin, David Meads, Rachael Kelley, Claire A. Surr

**Affiliations:** 1 Centre for Dementia Research, Leeds Beckett University, Leeds, LS1 3HE UK; 2 Academic Unit of Health Economics, Leeds Institute of Health Sciences, University of Leeds, Leeds, LS2 9JT UK

**Keywords:** Proxy-report, Care homes, Dementia, Inter-rater reliability

## Abstract

**Purpose:**

There are many validated quality-of-life (QoL) measures designed for people living with dementia. However, the majority of these are completed via proxy-report, despite indications from community-based studies that consistency between proxy-reporting and self-reporting is limited. The aim of this study was to understand the relationship between self- and proxy-reporting of one generic and three disease-specific quality-of-life measures in people living with dementia in care home settings.

**Methods:**

As part of a randomised controlled trial, four quality-of-life measures (DEMQOL, EQ-5D-5L, QOL-AD and QUALID) were completed by people living with dementia, their friends or relatives or care staff proxies. Data were collected from 726 people living with dementia living in 50 care homes within England. Analyses were conducted to establish the internal consistency of each measure, and inter-rater reliability and correlation between the measures.

**Results:**

Residents rated their quality of life higher than both relatives and staff on the EQ-5D-5L. The magnitude of correlations varied greatly, with the strongest correlations between EQ-5D-5L relative proxy and staff proxy. Internal consistency varied greatly between measures, although they seemed to be stable across types of participants. There was poor-to-fair inter-rater reliability on all measures between the different raters.

**Discussion:**

There are large differences in how QoL is rated by people living with dementia, their relatives and care staff. These inconsistencies need to be considered when selecting measures and reporters within dementia research.

At least 70% of care home residents in the UK [1] and over 50% of nursing home residents in the USA live with dementia [2]. As over half of residents die within 1 year of moving into a care home [3] and there is currently no cure for dementia, ensuring that individuals living with the condition maintain their quality of life is a priority within care homes. Quality of life (QoL) is often a key outcome measure in research studies evaluating the effectiveness of interventions in care home settings [4]. Additionally, changes in QoL have been examined as an indicator of the progression of dementia; studies show residents with a higher dependency on staff generally have a lower quality of life [5]. Measuring QoL has multiple important uses for clinical practice and research in care home settings; it is therefore critical to have accurate and validated tools to measure it in people living with dementia.

QoL is a subjective construct and participants usually rate their own experiences using self-report outcome measures. People living with dementia in care settings may have difficulties with communication, reasoning and recall accuracy [6], thus proxy informant outcome measures are commonly used alongside, or instead of, self-rated measures [7]. However, the use of proxy measures raises several issues with regard to accuracy. It is well established that proxy raters assess QoL lower than self-rated QoL within dementia research [8], since these two groups may have different concepts of QoL [9]. Correlation between scores on QoL measures specifically designed for people with dementia are generally low-to-moderate between self-, staff-proxy and relative-proxy, suggesting poor agreement [10, 11] and it is unclear who is the more accurate reporter [12]. The difference between proxy- and self-rated QoL is greater for individuals with higher levels of impaired cognition [10]. These proxies also rate the QoL of people with dementia as lower when they (the person with dementia) are experiencing more distress [10]. Evidence demonstrates that some people with dementia may overestimate their quality of life, suggesting a ceiling effect [8]. For example within one study, almost 50% of residents rated themselves as having the highest possible quality of life [12]. An additional issue for intervention research is the inability to blind proxies to treatment allocation in some studies, particularly when evaluating psychosocial interventions. Furthermore, the relationship between the proxy and resident, as well as time spent with the person during the reporting period, may influence proxy ratings [13]. These factors may affect measurement accuracy, increasing the chance of reporting errors. Despite these issues, there has been little examination of the relationship between different proxy raters across multiple measures.

Clinical trials rely on accurate outcome measurements and therefore, frequently collect data using multiple measures and multiple raters. A wide range of QoL measures exist for use with individuals with dementia, disease-specific (i.e. QUALID, DEMQoL) and generic (i.e. EQ-5D-5L). There are differences in these measures, in terms of their conceptualisation of QoL and procedures around administration or scoring [14]. Additionally, there is no recommended or standardised set of QoL measures for use in clinical trials of psychosocial interventions. One recent review identified five different dementia-specific QoL measures used by clinical trials in the past 10 years [15], making comparison of results between trials difficult. To address measurement issues and potential bias, researchers often choose to use more than one QoL measure within a research project [16], typically utilising a combination of self-rated and proxy-rated measures as appropriate.

Despite the widespread use of various QoL measures in dementia research, there has been limited comparison between self-report and staff proxy measurements in people living with dementia in care homes, where proxy-reporting is often relied upon [17]. Generally, low agreement between self-reported and staff proxy-reported QoL has been found, with mean resident-reported scores on the EQ-5D-5L [11] and QOL-AD [18] higher than those of staff proxies. The current evidence base, however, provides limited evidence of how ratings vary between different proxy-reporters and different measures, or how measures capture changes in QoL over time. To date, psychometric evaluation has usually examined differences between raters on a single QoL measure, limiting understanding of which QoL measures might be most appropriate, or how different measures compare for a single rater. Consequently, there is little consensus around the optimal way to measure QoL for people living with dementia [18]. Given QoL is one of the most frequent outcome measures used in dementia-related clinical trials, research on the relationship between measures is required to support selection of the most appropriate outcomes and raters, potentially leading to increased quality in outcome measurement.

To address this issue, this study examined aspects of validity and reliability, and relationships between four QoL measures across a large care home-based sample of three different raters (self-, staff proxy- and relative proxy-reporters). The aim of the study was to demonstrate how these differ, to allow researchers to consider which measure(s) and rater(s) is/are most appropriate for their research questions.

## Method

### Participants and procedure

Participants living with dementia were recruited from 50 care homes as part of a randomised controlled trial (for further details see [19]). Residents were eligible to participate if they lived in the care home permanently (i.e. were not receiving respite care), had a formal diagnosis of dementia or scored ≥ 4 on the Functional Assessment Staging of Alzheimer’s disease (FAST) [20]. They were ineligible if they had been formally admitted to an end-of-life care pathway or were mainly cared for in bed. For each participant, a staff proxy was recruited, who was eligible if he/she knew the resident well and had a permanent contract with the care home. The staff proxy was usually the resident’s key worker (i.e. a member of care staff). Where possible, a relative or friend of each person living with dementia was also recruited. The only eligibility criterion for relatives and friends was that they visited at least once every 2 weeks. All participants required sufficient proficiency in English to complete the measures.

Ethical approval was granted by the Bradford Leeds National Research Ethics Service Committee and Leeds Beckett University research ethics committee.

As part of the trial data collection, four QoL outcome measures were completed for each resident. Data collection took place over a two-week period. Proxy-reporters were asked to complete measures reflecting on QoL either ‘today’ or over ‘the past 2 weeks’ dependent on measure instructions; therefore, if they had not spent time with the person with dementia during this time, the research team sought another proxy. Some participants with dementia did not complete all measures, most frequently due to feeling too tired to continue.

### Quality-of-life measures

#### EQ-5D-5L [21]

The EQ-5D-5L is a five-item general (non-disease-specific) QoL measure that covers five dimensions: usual activities, mobility, self-care, anxiety/depression and pain. Respondents rate each item in terms of the level of problem they have with this domain (no problems, slight problems, moderate problems, severe problems and unable to complete task). An index score is calculated, from − 0.281 to 1, where higher scores indicate higher QoL, using health state valuations provided by country-specific general populations. This measure was completed by people living with dementia, staff proxy-reporters and relative/friend proxy-reporters. The EQ-5D-5L has been used with people with mild-to-severe dementia; however, there are concerns about its validity amongst this latter population [12].

#### DEMQOL proxy [22]

The DEMQOL proxy is a disease-specific QoL measure for people with dementia. It consists of 32 items that measures six domains of general health, mood, behavioural symptoms, cognition and memory, and physical and social functioning. Items are rated on a four-point Likert scale ranging from ‘a lot’ to ‘not at all’, with higher scores indicating higher QoL (five items are reverse scored). Scores range from 31 to 124. This measure was completed by staff proxy-reporters and relative proxy-reporters.

#### QOL-AD Nursing Home [23]

The QOL-AD Nursing Home version is a disease-specific 15-item questionnaire designed to measure QoL for people living with dementia in care homes. It covers areas including mood, relationship with friends and family, and physical condition. There are wording changes from the original QOL-AD to increase the relevance of the measure to people living in care homes, such as removal of an item around marital status and the addition of items related to relationships with staff and ability to make choice in daily life. Completed via self-report, this questionnaire uses simple language with four response options that are consistent across all items (poor, fair, good or excellent). Items are rated on a four-point Likert scale, with higher scores indicating higher QoL. Scores range from 15 to 60. Residents with mild-to-moderate dementia are reported to be able to self-rate QoL using this measure [18].

#### Qualid [24]

The QUALID is a disease-specific 11-item proxy-rated measure that rates both the presence and the frequency of indicators of QoL during the past 7 days. The measure covers 11 behavioural areas thought indicative of positive and negative QoL. A five-point Likert scale captures the frequency of each item, with total scores ranging from 11 to 55, with higher scores indicating higher QoL. This measure was completed by staff and relative proxy-reporters.

### Measure of functioning

#### FAST [20]

The Functional Assessment Staging of Alzheimer’s disease (FAST) measures the functional severity of dementia and was completed by a researcher with the care home manager. The tool is rated from 1 (no dementia) to 7 (severe dementia), with additional sublevels for 6 and 7 (a–e). To be eligible to participate in the present work, individuals were required to have a FAST score of 4 or above. This tool was completed to ensure that those without a formal diagnosis but who were still eligible could be recruited and was used to provide an understanding of sample demographics.

### Missing data

A researcher completed measures with each participant (except for some relative/friend proxy measures that were completed via post); therefore, the frequency of missing data at the participant level was extremely low, less than 1% for any one participant measure. Where some items were missing from a measure, this was dealt with by imputing the participant-specific mean item score in line with guidance [25]. Where a measure was not completed at all, this was marked as missing and not included in analyses. Therefore, different numbers of participants completed each measure and is highlighted where relevant.

### Data analysis

Data were analysed using SPSS v24. Correlations between measures were conducted to investigate concurrent validity, and correlations between assessors were conducted to establish inter-rater reliability. Spearman’s correlations were conducted between each of the measures, for self-report, staff proxy and relative/friend proxy, to establish whether significant correlations existed between the measures, and the magnitude of these. To calculate the internal consistency of measures, Cronbach’s alpha was conducted.

Inter-rater reliability was conducted between rater type on each of the QoL measures that were completed by at least two raters, using the weighted Cohen’s Kappa statistic with linear weights. The strength of the relationship was investigated to establish the level of agreement between raters over and above chance (ranging from − 1 to + 1) based on guidance [26]. The strength of the relationship is represented in the Cohen’s Kappa statistic as follows: values ≤ 0 indicate no agreement, 0.01–0.20 as poor agreement, 0.21–0.40 as fair, 0.41–0.60 as moderate, 0.61–0.80 as good and 0.81–1.00 as almost perfect agreement.

## Results

A total of 726 resident participants were recruited (see Table 1 for demographics) from 50 care homes, of which 377 completed self-report measures. Most participants were female (536; 74%), identified as White British (702; 96%) and had an average age of 85 (range 57–102). A staff proxy was recruited for each individual alongside 197 relatives/friend proxies.

**Table 1:** Participant demographics

Characteristics	
Age at registration (years) M(SD)	85.6 (7.64)
Gender	
Female	536 (73.8%)
Male	190 (26.2%)
Length of stay in care home (years) M(SD)	2.3 (2.34)
Ethnicity	
White British/European	702 (96.7%)
Other	24 (3.3%)
Funding type	
Local authority	352 (48.5%)
Self-funded	289 (39.8%)
Local authority and self-funded	34 (4.7%)
Continuing Healthcare	48 (6.6%)
Missing	3 (0.4%)
FAST	
1–3	6 (1%)
4	95 (13.6%)
5	74 (10.6%)
6	380 (54.5%)
7	142 (20.4%)
Missing	29 (3.9%)
QoL measures (baseline scores)	M (SD) N
EQ-5D-5L (self-report)	.868 (.18) 365
EQ-ED-5L (staff proxy)	.668 (.23) 723
EQ-ED-5L (relative proxy)	.520 (.25) 162
QUALID (staff proxy)	20.5 (6.95) 726
QUALID (relative proxy)	22.0 (7.18) 163
QOL-AD (self-report)	42.54 (6.31) 344
DEMQOL (staff proxy)	104.16 (9.77) 714
DEMQOL (relative proxy)	98.93 (14.30) 152

### Correlation between measures

There were significant correlations between most measures across different reporters (see Table 2). For relative proxy-completed measures, QUALID correlated with all other measures, EQ-5D-5L correlated with all measures except DEMQOL staff proxy and DEMQOL correlated with all but one (EQ-5D-5L staff proxy) measures. For self-report measures, EQ-5D-5L correlated with all other measures and QOL-AD correlated with all self-report and relative proxy-completed measures, but only QUALID of the staff proxy-completed measures. The magnitudes of these correlations varied, with the strongest correlations between EQ-5D-5L relative proxy with EQ-5D-5L staff proxy (.60) and self-report (.45), and QUALID staff (.42) and relative (.48) proxies.

**Table 2:** Correlations between measures—*N* participants provided in brackets

	EQ-5D-5L (self-report)	EQ-5D-5L (staff proxy)	EQ-5D-5L (relative proxy)	QUALID (staff proxy)	QUALID (relative proxy)	QOL-AD (self-report)	DEMQOL (staff proxy)	DEMQOL (relative proxy)
EQ-5D-5L (self-report)	–	.18** (377)	.45** (81)	.11* (377)	.33** (79)	.30** (329)	.12* (372)	.39** (75)
EQ-5D-5L (staff proxy)	.18** (377)	–	.60** (166)	.33** (691)	.27** (164)	.09 (336)	.11* (713)	.05 (152)
EQ-5D-5L (relative proxy)	.45** (81)	.60** (166)	–	.42** (160)	.48** (162)	.31* (68)	.07 (164)	.29** (150)
QUALID (staff proxy)	.11* (377)	.33** (691)	.42** (160)	–	.50** (159)	.11* (325)	.48** (680)	.20* (148)
QUALID (relative proxy)	.33** (79)	.27** (164)	.47** (162)	.50** (159)	–	.28* (67)	.28** (162)	.51** (150)
QOL-AD (self-report)	.30** (329)	.09 (336)	.31* (68)	.11* (325)	.28* (67)	–	.08 (332)	.42** (63)
DEMQOL (staff proxy)	.12* (372)	.11* (713)	.07 (164)	.48** (680)	.28** (162)	.08 (332)	–	.37** (150)
DEMQOL (relative proxy)	.39** (75)	.05 (152)	.29** (150)	.20* (148)	.51** (150)	.42** (63)	.37** (150)	–

*p < .05, **p < .001

There were a greater number of staff and relative proxy ratings overall, potentially as a function of scores not being completed by residents with more severe cognitive impairment. To explore whether this impacted the pattern of findings, mean staff and relative proxy ratings for EQ-5D-5L index scores were compared only for residents who provided an equivalent self-rating. When both scores related to the same group of residents, self-report scores (.78) remained higher than staff proxy-completed scores (.53), and self-report scores (.69) also remained higher than relative/friend proxy-completed scores (.41).

### Internal consistency

Internal consistency varied greatly between measures, although it seemed to be stable across types of participants (see Table 3). The DEMQOL (staff and relative/friend proxies) had good to excellent internal consistency (0.8–1.0), the QOL-AD (self-report) had good internal consistency (0.8–0.9), the QUALID (staff and relative/friend proxies) had acceptable internal consistency (0.7–0.8) and ED-5D-5L (all participants) had questionable internal consistency (0.6–0.7) [27].

**Table 3:** Cronbach’s alpha values for self- and proxy-completed measures by type of participant

	Self-report	Staff proxy	Relative proxy
EQ-5D-5L	.69	.62	.67
QUALID	–	.74	.72
QOL-AD	.86	–	–
DEMQOL proxy	–	.83	.91

### Inter-rater reliability

Agreement between the staff proxy-rated (*M* = 22.46) and relative/friend proxy-rated (*M* = 22.18) QUALID ratings (*N* = 159) indicated that although the level of agreement was above chance, there was a fair level of agreement between raters (*k *= .306 *p *< .001; see Table 4). Agreement between staff proxy (*M* = 101.99) and relative/friend proxy (*M* = 98.97) DEMQoL ratings (*N* = 150) was similarly above chance with poor/fair level of agreement between raters (*k *= .205, *p *< .001).

**Table 4:** Inter-rater reliability within QoL measures: Cohen’s Kappa

	EQ-5D-5L (self-report) Desc.	EQ-5D-5L (self-report) Ind.	EQ-5D-5L (staff proxy) Desc.	EQ-5D-5L (staff proxy) Ind.	EQ-5D-5L (relative proxy) Desc.	EQ-5D-5L (relative proxy) Ind.	QUALID (relative proxy)	DEMQOL (relative proxy)
EQ-5D-5L (self-report) Desc.			.121**		.170**			
EQ-5D-5L (self-report) Ind.				.004		.040**		
EQ-5D-5L (staff proxy) Desc.					.323**			
EQ-5D-5L (staff proxy) Ind.						.030**		
QUALID (staff proxy)							.306*	
DEMQOL (staff proxy)								.205*

*p < .001, **p < .0005

Agreement between ratings on the EQ-5D-5L descriptive and Index scores was also explored. There was fair agreement between staff proxy (11.47) and relative/friend proxy (14.14) ratings (*n *= 166) on the descriptive scale (*k *= .323 *p *< .0005). However, there was poor agreement between the 377 cases of staff proxy (10.22) and resident (7.46) ratings (*k *= .121 *p *< .0005). Similarly, in the 80 cases where relative/friend proxies (13.80) and residents (8.64) completed the EQ-5D-5L, there was poor agreement between ratings (*k *= .170 *p *< .0005).

Agreement between the Index EQ-5D-5L ratings was computed using the Cohen’s *k* statistic. As with the descriptive score, there was low agreement between resident and relative/friend ratings *k *= .04 *p *< .0005. There was very low agreement between resident and staff proxy ratings of Index QoL that was not statistically different to chance (*k *= .004 *p* = .649) and there was low agreement between the staff and relative/friend proxy ratings (*k *= .030 *p *< .0005).

Additionally, examination of the EQ-5D-5L by domain was conducted, to establish where differences between raters existed. Given this is a 5-item measure, large discrepancies between raters on a single item produce larger impacts on the overall score than for measures with more items. This demonstrated that on all domains, residents rated themselves as having ‘no problems’ more frequently than either relative/friend proxies or staff proxies (see Table 5). However, the difference was particularly large for self-care, where 76% of residents stated they had no problems with this whereas staff and relative/friend proxies rated that a much lower percentage of people were with no problems in this area (14% and 10%, respectively).

**Table 5:** Percentage of participants identifying problems or no problems across EQ-5D domains [*N*(%)]

EQ-5D dimension	Residents (*N* = 377)	Relative (*N* = 167)	Staff (*N* = 726)
Mobility			
No problems	248 (66)	31 (19)	269 (37)
Problems	129 (34)	136 (81)	457 (63)
Self-care			
No problems	285 (76)	16 (10)	100 (14)
Problems	92 (24)	151 (90)	626 (86)
Usual activities			
No problems	307 (81)	41 (25)	461 (64)
Problems	70 (19)	126 (75)	265 (36)
Pain/discomfort			
No problems	271 (72)	77 (46)	523 (72)
Problems	106 (28)	90 (54)	203 (28)
Anxiety/depression			
No problems	275 (73)	78 (46)	500 (69)
Problems	102 (27)	89 (54)	226 (31)

## Discussion

The present study compared QoL measures for people living with dementia across multiple measures with multiple raters. The inclusion of three raters allowed comparisons to be drawn between three groups of participants who may be recruited for clinical trials where QoL is an outcome. The internal consistency of measures varied from questionable (EQ-5D-5L) to good and excellent (DEMQOL), in line with previous evidence [see 28 for review]. The QOL-AD NH has been shown to have variable internal consistency [29, 30]; in the present study, however, we found the measure to have good internal consistency. This may be due to differing levels of cognitive impairment between samples, which may affect how reliably measures are completed. For example, a small sample of individuals with mild dementia was recruited for one study where the scale demonstrated good internal consistency [30], whereas a second study excluded those with advanced dementia [29]. Therefore, further research is required to establish when use of the QOL-AD NH is appropriate.

Correlations between different measures across different reporters were generally weak to moderate, in line with recent similar studies [11, 31]. This suggests that people living with dementia and those who support them do not perceive QoL in the same way, or that they may focus on different aspects of QoL, suggesting a need for several measures to be completed to ensure full coverage of perceived QoL. However, the issues may instead be due to differences in how QoL is conceptualised by people with dementia and by different types of proxy informant. This is especially important as proxy raters are thought to focus on issues such as pain and presence of neuropsychiatric symptoms, rather than QoL specifically [18]. Recent qualitative research suggests that staff members equate residents’ QoL with the quality of care delivered or the stage of their dementia, whereas relatives draw comparisons with the person’s QoL when they were younger, lived in their own home and did not have dementia [31]. It is unclear how people with dementia, particularly those who are care home residents, conceptualise their QoL compared to proxies, although those who are experiencing pain and have recently had a fall report lower QoL [18]. Future qualitative work should be undertaken to understand how QoL is conceptualised and reflected on by different types of participants when completing these measures.

Our findings broadly indicated an at best, fair agreement, between how the different raters perceived QoL for people living with dementia. The QUALID staff and relative/friend proxy ratings yielded the highest level of agreement, with fair agreement also found between staff and relative/friend proxies on the EQ-5D-5L. However, it is noted that fair agreement is not considered to be a reliable when establishing the validity of a measure, where a minimum value of .6 (substantial agreement) is recommended [32]. Notably, there was poor agreement between self-rated QoL and staff/relative proxy-rated QoL on the EQ-5D-5L. When the data were examined as Index values, agreement was not statistically above chance. This is in line with previous research, which has found discrepancies between people with dementia and their family members on this measure [33]. Further analyses, comparing the percentage of individuals who reported having problems in areas vs not having a problem in the area, revealed interesting discrepancies. Particularly, most residents reported no problems with self-care, whereas both staff and relative/friend proxies identified that most individuals had problems in this area. This may reflect additional issues with the sensitivity of this question, since people with dementia may feel uncomfortable or embarrassed stating they experience problems with self-care. Alternatively, care staff may overstate the problems individuals with dementia have, based on their own approach to provision of support for personal care, which may not be based on maximising independence, but rather on completing care tasks as efficiently as possible. Research should be conducted to explore these discrepancies in detail.

To date, a wealth of research studies have included multiple QoL and outcome measures but have not examined these systematically. For example, it has been highlighted that people with dementia are able to rate their QoL but that this differs from relative proxy ratings [34], without any exploration of why this might be. Other studies have stated that proxy ratings improve feasibility and should be used when people with dementia are unable to ‘answer by themselves’ to avoid having missing data [6], although this is presented without clear cut-offs to guide researchers. Therefore, researchers should be encouraged to examine the psychometric properties of the measures used within their studies, to help understand which are most appropriate for use with people living with dementia in care homes, with different degrees of cognitive impairment.

Previously, it has been stated that proxy-completed measures are the only option for individuals living with moderate to severe dementia [6, 9]. However, this fails to value the perspective and the insights into the QoL of individuals of people with dementia that may not be picked up by staff members, relatives or friends. Additionally, for research findings and any resultant policy changes to be meaningful, appropriate and valid QoL data must be collected [10] including recognition that people with dementia are able to provide meaningful commentary around their own QoL [7]. Researchers should, therefore, explore creative ways to work with those who struggle to communicate verbally to collect meaningful data. The burden of data collection for people with dementia needs to be considered within this, as some participants were unable to complete measures in the present study due to tiredness or boredom. Flexibility in researcher approach has been highlighted as important, providing participants with the opportunity to complete measures through several conversations or over 2 days if required [35]. Building relationships with participants with dementia can help to identify the best time of day for data collection, which could help increase the feasibility of self-completed data [35]. Furthermore, people with dementia have been shown to consistently rate their QoL higher than proxy raters. It is unclear whether this relates to an inability to accurately assess their performance or abilities against measure items or whether in fact proxies under estimate QoL based on their own, different perceptions of what is important. For example, people with dementia living in a care home may compare themselves with others living in the setting and may judge their QoL to be good comparatively or they may have reduced expectations about their own performance given their personal circumstances or may simply have a more positive outlook [34]. It is also noted that people living with dementia may benefit from overestimating their QoL, as a strategy of self-maintenance [36]. Further research is thus needed to assess why people with dementia living in care home settings make particular judgements on QoL items and what this means for assumed ‘accuracy’ and interpretation of results.

More widely, there are concerns about the quality of QoL measures in general and the feasibility of their use with people with dementia. Most of the existing dementia-related QoL measures have had limited psychometric evaluation [37, 38]. For example, to date, the relationship between the QOL-AD NH and any health-related outcomes has not been examined (criterion validity). Furthermore, the QUALID has demonstrated poor criterion validity, in both studies that have examined this [39, 40]. However, whilst these issues are concerning, in part, these may be due to methodological issues of the studies examining the psychometric properties of measures rather than highlighting underlying problems with the measures [4].

## Limitations

Although multiple measures were collected in the present study, only one measure was completed by residents that proxy-reporters also completed (EQ-5D-5L). Therefore, self- and proxy-rating comparisons could not be drawn from QUALID and DEMQOL. In future, where possible, the same measures should be completed by residents and proxy-reporters, in order to be able to draw more in-depth comparisons. Individuals who lived in the care home and were cared for in bed or were formally admitted to an end of life care pathway were not eligible to participate in the present study. These individuals may have been expected to have the lowest QoL and therefore the present study may not fully capture the breadth of QoL experienced by those living in care homes. Additionally, in line with previous evidence [9], those who completed self-report measures are likely to have been in the earlier stages of dementia and therefore have higher QoL than those in the later stages, which were not accounted for in the present study. Researchers need to develop alternative strategies to ensure that the perspectives of those with later-stage dementia are captured [35]. Furthermore, we do not understand why participants provided the rating that they did for each item. Collecting additional qualitative data to explore this issue would improve understanding around why differences in ratings exist.

There are demographic characteristics (for example in the qualifications and experience of staff, and relationships of relative/friends to residents) that may have affected QoL ratings [36]. For example, spouses have been found to rate QoL in people living with dementia as higher than adult children [33]. We did not stratify our analysis to explore within-group variations in QoL ratings due to poor completion of demographic details by these participants. Understanding predictors of variability in QoL ratings within relative/friend and staff proxy groups constitutes a valuable should be an on-going focus for future research.

Future research should enhance recent reviews [37, 41] and conduct a meta-analysis of different QoL measures completed by different raters over time, in order to establish which are most meaningful and suitable for use. One recent narrative review concluded that self-report and proxy-report DEMQOL and EQ-5D-5L should be used [15]. However, within the 41 studies reviewed, only four used DEMQOL; therefore, this conclusion is based on limited evidence. We found that DEMQOL had good internal consistency in the present study and scores on this measure significantly correlated with five of the additional seven measures, although these correlations were all weak, except for staff proxy-reported QUALID. In addition, QUALID correlated with all (relative/friend proxy-reporters) and all but one (staff proxy-reporters) measures. Although it demonstrated weaker internal consistency than both QOL-AD and DEMQOL, our results suggest that DEMQOL proxy may offer the most thorough and comparable measure of QoL; however, we did not collect self-report DEMQOL and cannot make a definitive judgment without this. A care home-specific version of the DEMQOL has recently been developed, in line with other measures such as the QOL-AD NH, which may provide further utility for this measure within care homes [42].

## Conclusion

In conclusion, measuring quality of life for people with dementia is complex and often involves multiple measures completed by multiple raters. Whilst it is acknowledged that self-report data are the optimal method of data collection, the limitations of this method are also widely reported, particularly as ability to complete measures is likely to decline over time for those with dementia. The low levels of agreement between relative/friend and staff proxy raters on these measures, however, bring into question the appropriateness of proxy-rated data within this population. Given that residents may overestimate their QoL, it is difficult for researchers to establish which measure provides the most reliable or valid report of individuals’ QoL. The lack of other viable alternatives at present means that researchers should be aware of these issues and interpret their data with caution. This study highlights the need for researchers and practitioners to better understand of the impact of rater choice on QoL outcomes. It is not possible to recommend staff or relative/friend proxy ratings as more or less accurate than self-ratings, as proxy rating may be biased by factors that unduly influence perceived QoL (such as self-care ability), whilst the same factors may have little impact on QoL as experienced by the participant or resident. Therefore, researchers need to give greater consideration of the influence of raters when selecting QoL outcome measures and their completion.
